# Effect of Different Reducing Agents on Aromatic Compounds, Antioxidant and Chromatic Properties of Sauvignon Blanc Wine

**DOI:** 10.3390/foods9080996

**Published:** 2020-07-24

**Authors:** Ana-Marija Jagatić Korenika, Josipa Biloš, Bernard Kozina, Ivana Tomaz, Darko Preiner, Ana Jeromel

**Affiliations:** 1 Department of Viticulture and Enology, Faculty of Agriculture, University of Zagreb, Svetošimunska 25, Zagreb 10000, Croatia; josipa.bilos@gmail.com (J.B.); bkozina@agr.hr (B.K.); itomaz@agr.hr (I.T.); dpreiner@agr.hr (D.P.); amajdak@agr.hr (A.J.); 2 Center of Excellence for Biodiversity and Molecular Plant Breeding, Svetošimunska 25, Zagreb 10000, Croatia

**Keywords:** antioxidant activity, aromatic compounds, chromatic parameters, OAV, reducing agents, ROC, Sauvignon Blanc, sulfites, white wine

## Abstract

Sulfur dioxide (SO_2_) is widely the most used enological additive with reductive, antiseptic and dissolving properties. According to increasing health concerns and the gradual decrease in total SO_2_ concentrations allowed in wine, alternative and supplementary agents for preservation are being investigated. For this reason, the current study was focused on the impact of different commercial reductive agents on white wine antioxidant activity and chemical composition. The effect of additives that combine sulfites, ascorbic acid and enological tannins were compared against standard 5% sulfurous acid (H_2_SO_3_) during the pre-fermentative treatments of Sauvignon Blanc must (*Vitis vinifera* L.). The basic parameters of quality, free amino-nitrogen and total polyphenoliccompounds in must were analyzed. Gas chromatography and spectrophotometric methods were used to investigate the overall volatile composition, antioxidant and chromatic parameters in wines. The obtained results undoubtedly pointed out the positive effect of sulfuric acid on the fermentation dynamics. Furthermore, application of combined reducing additives with potassium metabisulfite, L-ascorbic acid, gallotannins and ellagitannins, resulted in a higher antioxidant capacity and increased concentration of aromatic compounds and their odor activity values in Sauvignon Blanc wine.

## 1. Introduction

Sulfites or sulfiting agents such as sulfur-containing salts (sodium and potassium metabisulfite or bisulfite), sulfurous acid and sulfur dioxide (SO_2_) are the most utilized preservatives and seem indispensable in winemaking due to their antioxidative, antimicrobial and dissolving properties. While moderate oxidation improves the quality and sensory characteristics of red wines, SO_2_ is essential for the preservation of the color and aroma of white wines. Due to its positive conservation and regeneration effect on wine aroma, SO_2_ is the most effective additive in wine production [1]. Besides the direct oxygen scavenging and inhibition of oxidation enzymes, the main antioxidative function of SO_2_ is in its binding to hydrogen peroxide, which is the product of oxygen reduction. In that process, it prevents the aldehyde production and oxidation of other readily oxidizable compounds [2,3,4]. Moreover, SO_2_ also reduces quinones (brown polymers) back to their phenol form and improves polyphenolic wine composition [5]. Among its multifunctional properties, SO_2_ has a very important antimicrobial role against different unwanted microorganisms such as epiphytic yeasts, lactic acid bacteria (LAB) and, to a lesser extent, acetic acid bacteria [6].

In addition to being convenient and versatile, the excessive use of SO_2_ can have a detrimental effect on wine quality, including the neutralization of wine aroma, the formation of hydrogen sulfide, unwanted aromas and flavors and cloudiness after bottling [7,8]. Unfortunately, SO_2_ may cause a range of adverse clinical effects in sensitive individuals, from headaches, dermatitis, abdominal pain, diarrhea, asthma and bronchoconstriction [6,9]. As a commonly used preservative in low pH foods, such as juices and fermentable drinks, it is important to consider the cumulative effect on the consumers [10]. For these reasons, the International Organization of Vine and Wine gradually decreased the maximum permitted levels of total SO_2_ in wine [11]. In the European Union, the allowed limit for conventional wines is up to 150 mL/L in red ones and 200 mL/L in white and rosé wines [8]. A typical target for free SO_2_ to prevent wine oxidation is 20 to 40 mg/L [4], depending on wine style, aging conditions and expected shelf-life [12]. Contemporary trends that include a healthy lifestyle mean that consumers are looking for healthier and higher quality products. Due to increasing demands for low-sulfite or sulfite-free products, winemakers are facing one of the biggest challenges of modern winemaking, i.e., how to find healthier alternatives that could replace the positive properties of SO_2_ or to use it in combination with reduced doses of SO_2_ in order to protect the chemical and sensory properties of the wine.

In recent years, various groups of chemical, physical and natural methods have been proposed as promising tools for the replacement of SO_2_ and have been discussed in different reviews, mainly through antimicrobial properties [8,12,13,14]. Until today, only a few authors have analyzed the influence of some alternative antioxidants and their combined effect with SO_2_ on the white wine oxidation process, volatile composition and shelf life, mainly by addition prior to bottling [15,16,17]. As a strong antioxidant and reducing agent, more active with molecular oxygen, through the autooxidation, which generates dehydroascorbic acid and hydrogen peroxide (H_2_O_2_), ascorbic acid is used to complement the effect of SO_2_ [16]. The main disadvantage is affecting color development in white wines and use of SO_2_ as a complementary agent, which can ensure efficient scavenging of dehydroascorbic acid, H_2_O_2_ and its degradation products [16,18]. However, according to [16] its impact on oxidative wine aging is controversial and debatable. In order to improve chemical and sensory characteristics of white wine, plant extracts, like tannins have been investigated [15,17,19,20]. Hydrolysable tannins, such as gallotannins extracted from oak galls and ellagitannins from oak or chestnut, which are not naturally present in grapes, make up the most sold commercial tannins [19]. According to [15] the pre-fermentative addition of enological tannins can effectively influence the oxidative phenomena on white must and wine.

Color is one of the most important sensory characteristics of white wine and according to [21], it plays a greater role in defining perceived odor than the chemical constitution of wine. Color intensity and hue measurements give useful information about phenolic concentration and tendency for oxidation, especially for wines treated with alternative reduction agents [8]. Wine aroma is a rather complex feature, formed by aromatic compounds from grapes as well as from compounds formed during and after alcoholic fermentation. The aromatic properties of Sauvignon Blanc wines mainly arise from varietal thiols and methoxypirazine, while the esters, terpenes and other aromatic compounds play a supporting role, enhancing the complexity of the wine [22]. The majority of aromas are developed during the process of alcohol fermentation and storage via enzymatic or non-enzymatic esterification of carboxylic acids [4].

There are some studies about the influence of alternative agents compared to sulfites on white wine properties, added in different stages of vinification [1,15,16,17,23]. One of the main concerns regarding these studies is that they were conducted on a laboratory-scale and on model wines so there are insufficient data on how commercially available antioxidative alternatives affect the color and aroma of different white wine styles on a larger scale. Despite growing interest in the use of natural/organic preparations as potential alternatives to SO_2_ (phenolics or natural extracts), the results so far have been discouraging [24]. Therefore, this paper reports on the effects of different commercial reducing agents that complement SO_2_ with ascorbic acid and enological tannins, added in the early stages of the processing, i.e., before the alcoholic fermentation with the main purpose of preserving white wine aromas, color and prevention of oxidation.

## 2. Materials and Methods 

### 2.1. Wine Production

**Figure 1:**
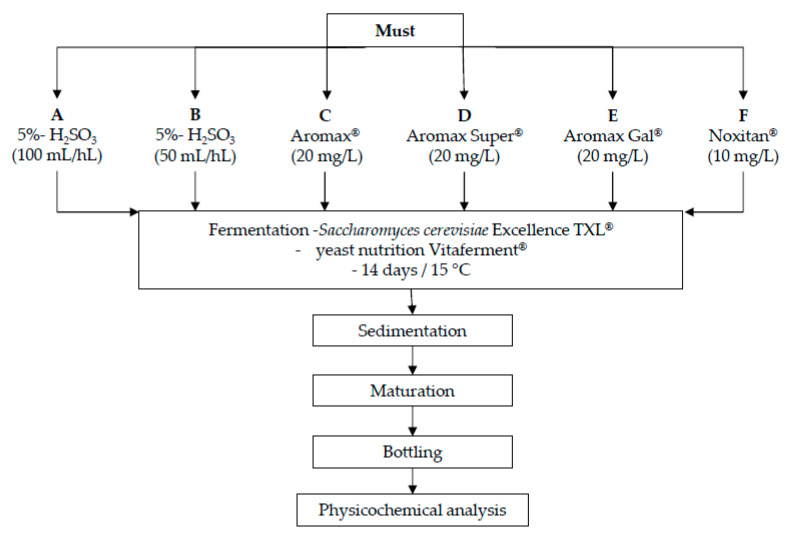
Scheme of the production of Sauvignon Blanc cv. white wine.

The grape variety used in this experiment was Sauvignon Blanc (*Vitis vinifera* L.), cultivated and processed at the Experiment Station of Faculty of Agriculture (growing hill Zagreb, Croatian Uplands). Experimental wines were produced by the processing of 450 kg of grapes. Manual grape harvesting was conducted on the 6th September 2018. The grapes were processed by using of automatic destemmer and crusher and pressing was done by a hydraulic press. Basic must quality parameters like sugar concentration, total acidity, pH, free α-amino nitrogen and total phenols were determined. The must was divided into six 50 L -stainless steel vessels (samples A, B, C, D, E, F) and different reducing agents were added to each sample (Figure 1). The reductive addition treatments were: (A) SUMPOvin (Inovet doo, Varaždin, Croatia), a 5%-sulfurous acid used in the standard dosage 100 mL/hL; (B) SUMPOvin, 5%-sulfurous acid, 50 mL/hL, (C) Aromax^®^ (AEB, Brescia, Italy) combines 50% potassium metabisulfite (54 mg/L of SO_2_) and 50% L-ascorbic acid (60 mg/L); (D) Aromax Super^®^ (AEB, Brescia, Italy) 50% potassium metabisulfite, 35% L-ascorbic acid and 15% pure gallotannins; (E) Aromax Gal^®^ (AEB, Brescia, Italy) 50% of potassium metabisulfite, 35% of L-ascorbic acid and 15% of pure gallo- and ellagitannins; and (F) Noxitan^®^ (AEB, Brescia, Italy) combination of potassium metabisulfite (50 mg/L SO_2_) and ellagitannins. After 24 h of sedimentation, clear musts were separated from the sediment using closed pumping and were put into 12 glass containers of 25 L. Six types of Sauvignon wines were elaborated—each experimental variant was reproduced in two replicates for each one (n = 2). The grape musts were inoculated with *Saccharomyces cerevisiae* Excellence TXL^®^ yeasts (20 g/hL, Lamothe Abiet, Canejan, France) with activator of the yeast Oenostim^®^ (30 g/hL, Lamothe Abiet, Canejan, France). Complex yeast nutrition Vitaferment^®^ (Lamothe Abiet, Canejan, France) based on ammonium and vitamin B1 was added to help the yeast reproduction and decrease anomalies in the fermentation dynamics conducted at 15°C. At the end of fermentation, the basic enological analyses were done, and 5%-H_2_SO_3_ was added to correct the free SO_2_ concentration level at 25 mg/L. After approximately 60 days, each treatment was bottled and the wines were analyzed after 3 months of storage at cellar temperature (15–17 °C).

### 2.2. Must and Wine Analyses

#### 2.2.1. Physicochemical Analysis

Basic wine parameters including alcohol strength (% *v*/*v*), reducing sugar, total and volatile acids, extracts, ash, free and total SO_2_ and pH values of the given samples were analyzed using the methods prescribed by OIV [25].

#### 2.2.2. Free α-Amino Nitrogen

Free α-amino nitrogen (FAN) was determined by spectrophotometer Specord 400, (Analytik Jena, Jena, Germany), using the method proposed by Dukes and Butzke (1998) [26].

#### 2.2.3. Total Phenols

Total phenols (TP) were determined spectrophotometrically using the method by Singelton and Rossi [27] based on the color reaction of the phenolic compounds with the Folin–Ciocalteu reagent. The average of three measurements was used as the final absorbance value. The results were expressed in gallic acid equivalents (mg/L of gallic acid).

#### 2.2.4. Total Antioxidant Activity

The total antioxidant activity (AA) of the wines was evaluated using the ABTS-free radical method described by Re et al. (1999) [28] Absorbance measurements were transformed to antioxidant activity using Trolox as a reference. Absorbance measurements were recorded on a Specord 400 spectrophotometer. The cation radical ABTS^+^ is generated directly by the reaction of an ABTS stock solution (7 mmol/L) with 140 mmol/L potassium persulfite in a 1:0.5 stoichiometric ratio; the mixture was allowed to stand in the dark for 12 to 16 h. Next, 5 mL of the formed cation radical ABTS^+^ was mixed with 50 μL aliquots of wine and the absorbance was measured at 734 nm, 6 min after mixing. A blank control of an ethanol/water mixture was run for each assay. Results are expressed as μmol of Trolox equivalents/L of wine (TEAC/L). All determinations were carried out in triplicate.

#### 2.2.5. Color Parameters

Color intensity, hue/tint/tonality and pigments were analyzed by direct measurement of wine absorbance at 420, 520, and 620 nm by using a Specord 400 spectrophotometer (Analytik Jena, Jena, Germany). The color intensity (CI), color tint/tonality/hue (T), and the proportion of yellow (% Ye), red (% Rd), and blue (% Bl) pigments were calculated as follows: CI = Abs 420 + Abs 520 + Abs 620; T = Abs 420/Abs 520; % Ye = (Abs 420/CI) × 100, % Rd = (Abs 520/CI) × 100, % Bl = (Abs 620/CI) × 100 [29].

#### 2.2.6. Volatile Compounds Analysis

Analysis of wine volatile compounds was performed according to the method described by [30]. In brief, isolation of volatile compounds was done by applying solid phase extraction procedure on LiChrolut EN cartridges (200 mg/3 mL, Merck, Darmstadt, Germany). The GC analysis was performed on an Agilent 6890 system coupled with 5973 N mass spectrometer with a ZebronTM ZB-WAX capillary column (60 m × 0.32 mm i.d., with 0.5 μm film thickness, Phenomenal, Torrance, USA). The flow rate of helium was 1 mL/min. The mass spectrometer was operated in an electron ionization mode at 70 eV with selected ion monitoring (SIM) with selected ions. Compounds were first identified using NIST/EPA/NIH MS Search 2.0 and our own mass spectra libraries. Identities of most of them were then confirmed by comparison of their linear retention indices and EI mass spectra with those of reference compounds. Quantification of all examined compounds was done by the external standard method.

#### 2.2.7. Odor Activity Values and Relative Odor Contributions

Odor activity values (OAV) and relative odor contributions (ROC) are two conventional indicators used to estimate the sensory contribution of the aromatic compounds to the overall flavor of wines. OAV is calculated as the quotients of their concentration (c) and the corresponding odor perception threshold (t) reported in the literature [31]. Aromatic compounds with OAV > 1 can contribute to the overall aroma of wine [32]. The ROC of each aroma compound is calculated as the ratio of the OAV of the respective compound to the total OAV of each wine [33].

### 2.3. Statistical Analysis

Mean values of concentrations and their standard deviations were calculated from three replicates. One-way analysis of variance (ANOVA) was performed using the SAS System for Windows 9.0, 2004 (SAS Institute Inc., USA). The differences in the content levels were estimated by t-test. The probability of *p* ≤ 0.05 was considered statistically significant. Multivariate analysis was carried out with XLSTAT software v.2020.3.1. (Addinsoft, New York USA). The results of Principal Components Analysis (PCA) presented as two-dimensional PCA plots were used to identify the differences between wines.

## 3. Results and Discussion

### 3.1. Must Composition

After the primary processing of grapes, the concentration of sugar (95 °Oe), total acidity (7.4 g of tartaric acid equivalents/L), free α-amino nitrogen (28.49 (FAN)/mg/L), total phenols (340.35 (TP)/mg/L) and pH value (pH 3.3) in the sample of Sauvignon blanc must were determined. A relatively high sugar concentration determined in the must indicated a higher alcohol percentage > 13 vol. % in the final product. Sauvignon Blanc must contained a high amount of total acidity and the pH value was within the range that is optimal for white wines (from 3.1 to 3.4) [34]. The free α-amino nitrogen (FAN) concentration of the must indicated that the grape must have needed to be supplemented with yeast nutrients, which provides the regular course of the alcoholic fermentation.

### 3.2. Physicochemical Properties, Total Phenols and Antioxidant Potential of Wines

The results of basic physicochemical analysis of wines are shown in Table 1. According to the sugar concentration of the must, concentrations of alcohol were in the range 13.5–13.7%, *v*/*v*. Only the samples A and B fermented to dryness (< 4 g/L of residual sugar), while all the other treatments resulted in semi-dry wines. Due to the fact that the same type of base wine and the same yeast were used, the difference in the fermentation dynamics can be partially prescribed to the different reducing agents used. Sugar-free extract was in the range of 17.7–18.9 g/L, while the highest value was registered in sample B. Extract is a very important component of the wine’s quality, which hugely improves its fullness and harmony and the range for dry white wines is usually below 25 g/L [35]. The total acidity (TA) was between 5.8 and 6.6 g/L. The highest concentration was measured in sample B, while the were significantly lower in samples A and D. Volatile acid concentration was between 0.54 and 0.70 g/L, which is in accordance with the values prescribed in the Regulations of the Wine Production [36]. Considering the fact that the use of sulfites in different dosages and combinations is the main subject of this work, it is important to note that the results of the free, bound and total SO_2_ in wines after the fermentation showed the expected absence of the free SO_2_. Bound SO_2_ ranged from 37 to 59 mg/L, while the concentration increased in the following way: A < B < F < C < D < E. The total SO_2_ concentration met the regulations prescribed in the Regulations of the Wine Production [36].

According to other studies, final results of TP (Table 1) were within the range of 92–482 mg/L found in white wines from Croatia [37,38,39]. There were significant differences in TP values among all samples, from the lowest concentration in sample A to the highest in sample E. Similar values for TP, ranging from 191–248 mg/L, were found in other Sauvignon Blanc wines [22]. Contrary to other research [24,40], a high concentration of TP was found in wines with a higher total SO_2_ concentration, which may have prevented phenolic oxidation. Results highlighted the lowest ABTS and TP values in wines with 5% H_2_SO_3_ added in both doses (A and B) and in wine F (potassium metabisulfite + ellagitannins). Significantly high antioxidant activity was detected in wines with a high concentration of bioactive phenolic compounds that were treated with reducing agents containing ascorbic acid and tannins (C, D and E).

### 3.3. Chromatic Properties

**Figure 2:**
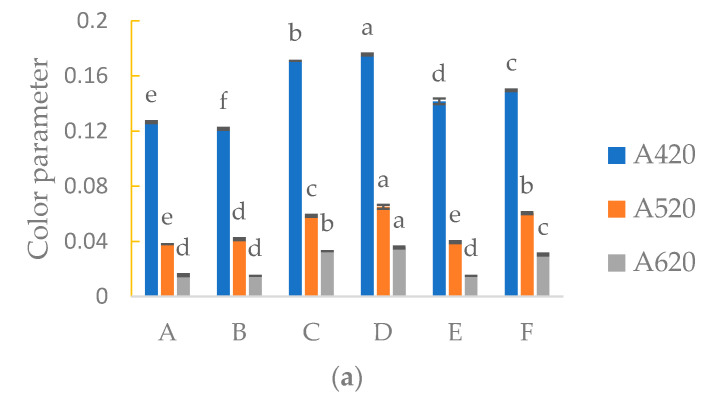

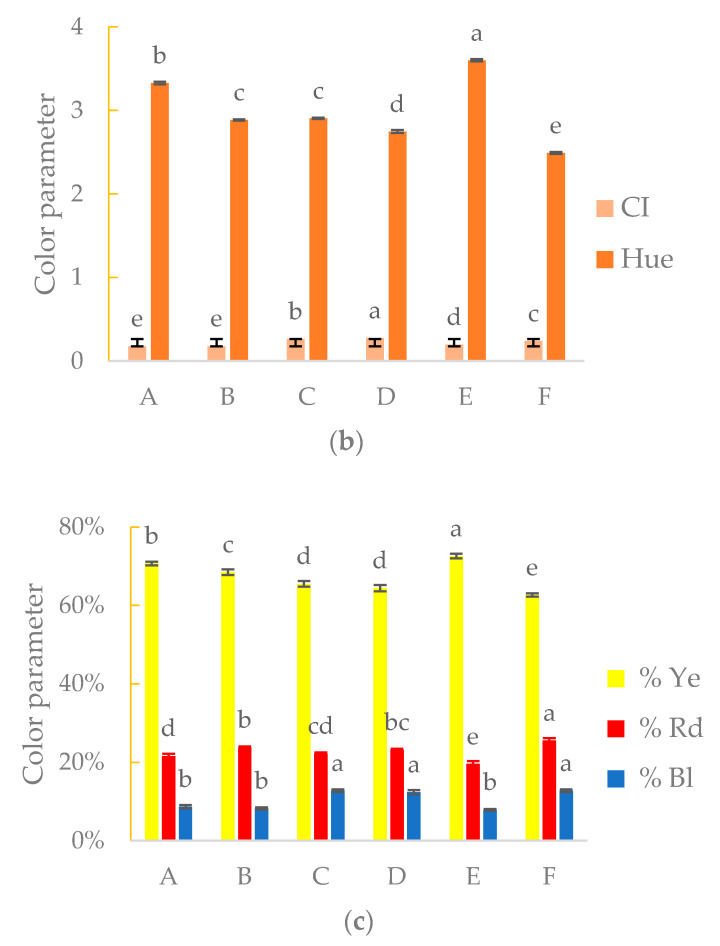
Absorbance at 420, 520 and 620 nm; (**a**), color intensity (CI) and color hue values; (**b**) proportion of yellow (% Ye), red (% Rd) and blue (% Bl) for Sauvignon Blanc wines; (**c**) columns marked with different letters (a, b, c, d, e, f) differ significantly (*p* ≤ 0.05) among treatments.

Figure 2 shows the results of color evaluation in terms of color intensity (CI), hue value (T) and proportion of yellow (% Ye), red (% Rd) and blue (% Bl) pigments. White wines transmit essentially all wavelengths of light to a high degree (around 80%), but then absorb strongly in the short wavelength end of the spectrum. The absorbance values on 420 nm are conventional reference absorbance values for the evaluation of white wine color (Figure 2a). Thus, they are absorbing blue light while transmitting other wavelengths resulting in their typical yellow color [41]. Significantly higher absorbance at all wavelengths, especially at 420 nm, was noticed in samples C and D, together with highest color intensity. The low absorbance was in samples A and B, which had lower concentrations of total SO_2_, total phenols and antioxidant activity. These results are in agreement with [15], who observed an increase in optical density at 420 nm when studying the substitution of SO_2_ by lysozyme. A significantly high proportion of yellow was detected in sample E with a high concentration of TP, antioxidant activity and total SO_2_. A significant decrease in hue value was observed in all samples compared to sample E (*p* < 0.0001). There was no direct correlation between concentration of total SO_2_ and color parameters in other wine samples.

### 3.4. Aroma Compounds

Besides the basic enological properties, 92 volatile compounds divided into 7 chemical groups were analyzed (Table 2). The highest number of compounds was in the group of alcohols (24), followed by esters (21), monoterpenes (17), volatile phenols (8), aldehydes (5), fatty acids (5) and miscellaneous compounds (9). The concentration of the total aromatic compounds increased following the succession: A < D < C < F < E < B. The most represented group of the individual compounds were the alcohols with highest total concentration in samples B and E. According to [15], SO_2_ had a significant influence on alcohol production that was not completely confirmed by actual study. In this research, the total concentration of the alcohols was in the range of 99.45–127.94 mg/L, which still has a positive impact on the wine aromas and enhances aromatic complexity according to [42]. The most abundant compound related to whiskey, solvent or nail polish aroma was isoamyl alcohol. The alcohols that could contribute to the wine aroma with concentrations above their sensory perception threshold were phenyl ethyl alcohol with rose notes (the highest concentration being in samples B and E), 2-methyl-1-butanol associated with black truffle (with no statistical difference among all samples) and 1-hexanol with a freshly cut grass aroma (the highest concentration in samples B and A) [43]. The highest concentration of fruity esters was found in sample D, while the lowest was found in sample A. The tannins seemed to have the most positive influence on ester production, which is in accordance with [15]. As suggested in other papers, these results may be due to the ability of tannins added before fermentation to affect the presence of oxygen in musts and wines, as a consequence of a double mechanism of enzyme inhibition and of radical-scavenging activity. Tannins can quickly drop the oxygen availability, contributing to preserve the ester amounts in wines [44]. The most representative ester in all samples was ethyl-hydrogen succinate. Esters of the succinic acid are used to imitate the smell of butter, rum, brandy, grapes and raspberries [45]. The second most representative ester was isoamyl acetate with a banana scent, then diethyl succinate with an apple aroma, ethyl octanoate with the scent of pears and ethyl decanoate with sweet fruity aroma. According to [39], the most abundant ester in white wines from Croatia, after ethyl acetate, was isoamyl acetate with a concentration between 0.31–4.14 mg/L, then ethyl hexanoate and ethyl octanoate. A study on Sauvignon Blanc wines by [22] reported that ethyl acetate, isoamyl acetate and ethyl hexanoate were dominant esters.

The group of monoterpenes is representative of grape variety aroma profiles. The most common compound was nerol, with the highest concentration in samples B and D, and geraniol in sample D. The aroma of nerol can be described as flower/citrus, while the geraniol can be described as rose. This research showed different results compared to the results of the indigenous white varieties in Croatia, where the most representative monoterpene was linalool with a citrus-flowery aroma [39]. β-damascenone, a representative of C13-norisoprenoid, with its flowery aroma that improves the aroma of Sauvignon Blanc wines, was also analyzed. The concentrations were above its threshold with the highest concentration in sample F. The most abundant compound in the group of volatile phenols was tyrosol (11–23 mg/L), a natural antioxidant present in wine, as the product of the yeast metabolism. The highest concentration was in sample A. Tyrosol concentrations above 25 mg/L in still wines can cause a bitter taste [46]. The second most-represented compound was the compound with a pleasant jasmine and almond taste—benzyl alcohol, with the highest concentrations found in samples A and B. The fatty acid with the highest concentrations was propionic acid, which was quantified in sample D. All the fatty acids analyzed in this research were present in the concentrations below the sensory perception threshold that usually has a positive impact on aroma complexity. The highest concentration of the total aldehydes was in sample D, while the most abundant compound was 2-octenal with its green-nutty aroma.

**Figure 3:**
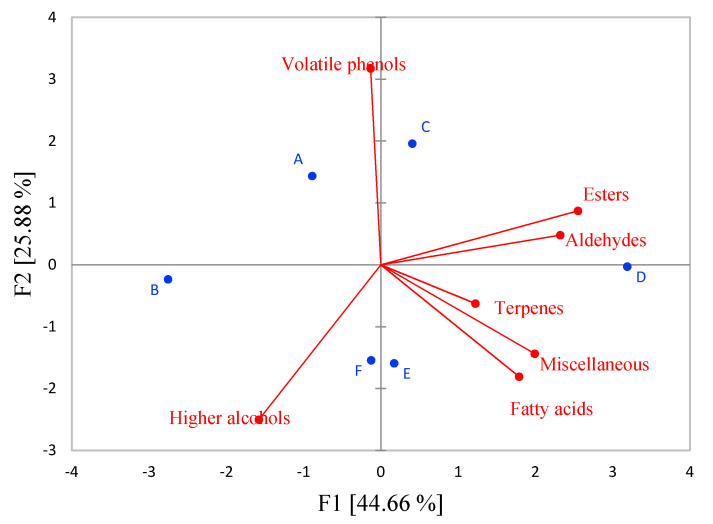
Distribution of the wine samples in a two-dimensional coordinate system defined by the first two principal components (PC1 and PC2) according to the applied reducing agents (A, B, C, D, E, F) and groups of aromatic compounds.

In order to evaluate the effect of applied reducing agents on the groups of the aromatic compounds in Sauvignon Blanc wines, a PCA analysis was conducted as well. The results of PCA are shown in Figure 3, where the first two components explained 70.54% of the total variance. A clear separation of the analyzed samples in a two-dimensional coordinate system was evident. Wines treated with 5% H_2_SO_3_ (A and B) and potassium metabisulfite/ellagitannins (F) were clearly separated by PC1 from wines treated with reductive agents that include ascorbic acid (Figure 3). Pre-fermentation treatment with H_2_SO_3_ and potassium metabisulfite/ellagitannins was characterized with total higher alcohols and volatile phenols. Wines produced with reductive agents that include ascorbic acid were discriminated by the majority of aromatic compound groups analyzed. It was supposed that sample D, located at the rightmost of PC1, was positively correlated with total esters, aldehydes and terpenes.

### 3.5. Odor Activity Value (OAV) and Relative Odor Contribution (ROC)

To evaluate the effects of individual aromatic compounds on overall aroma profile of Sauvignon Blanc wines, OAV values and ROC indexes were calculated. Each aromatic compound has been associated with an odor descriptor as reported in the literature (Table 3). Only 17 compounds out of 92 exceeded the threshold values (OAV > 1). In sample D, 2-octenal (OAV = 942.40) had the highest OAV among all compounds, more than two times higher than in samples A and B. Together with hexanal (OAV = 103.75 in B) it has an enhanced green and grass aroma, common descriptors of Sauvignon Blanc wines. ß-damascenone had the second highest OAV (148.80) in sample F. Isoamyl acetate had the highest OAV (76.01) in sample D, again more than two times higher than in sample B. Among terpenes, only geraniol and linalool had an OAV > 1, both with a flowery-citrus aroma, with high values in samples A and B. The higher alcohol, i.e., 1-octen-3-ol, which had the highest OAV in samples B (44.70) and A (39.36), is associated with a mushroom aroma. The highest OAV (2.42) of rose-like phenylethyl alcohol was noted in sample E. The same number of the highest OAVs was found in samples D and B, mostly from fruity esters, but the highest OAV in total was noted for sample D. On the basis of the ROC index, a further contribution of each individual compound on the wine aroma was analyzed (Table 3). Although the most abundant compounds with the highest OAVs were found in wine sample D, some of them, like 2-octenal and ethyl hexanoate, ethyl octanoate and ethyl butanoate displayed greater relative odor contribution (ROC) in samples C and A. Phenylethyl alcohol and ß-damascenone had the highest ROCs in sample B, even though their OAVs were the highest in samples E and F, respectively.

**Figure 4:**
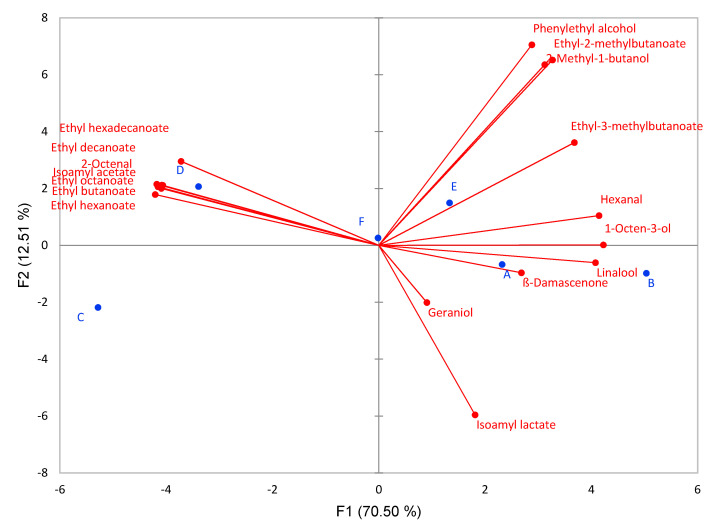
Distribution of the wine samples in a two-dimensional coordinate system defined by the first two principal components (PC1 and PC2) according to the applied reducing agents (A, B, C, D, E, F) and aromatic compounds with OAV > 1 in Sauvignon Blanc wines.

To further define the aromatic compounds influencing the wines, OAV data were used for PCA analyses (Figure 4). Variables included in the PCA were limited to OAV > 1 (Table 3). As it can be seen from Figure 4, the first principal component (PC1), accounting for 70.50% of the total variance, differentiates samples C and D from the other four samples, while PC2 that explains 12.51% of variance separates samples A, B, C from D, E and F. In the loading plot shown, most varietal aromas like linalool, geraniol and ß-damascenone were located on the right side, positively linked with PC1, indicating that the addition of H_2_SO_3_ resulted in a higher sensory contribution of these aromatic compounds. On the other hand, PC2 contributed to the differentiation of wines D and E from the other samples. Wine D treated with potassium metabisulfite, ascorbic acid and gallotannins, on the negative side of PC2, was characterized with higher contributions of most ethyl esters and therefore, fruity aromas. The PCA analysis shows that the treatment of wine E with a combination of potassium metabisulfite/ascorbic acid/gallo- and ellagitannins, increased the sensory contribution of higher alcohols like phenylethyl alcohol with a floral aroma.

## 4. Conclusions

Pre-fermentative treatments with different reducing agents had a significant effect on the physicochemical, antioxidant, aromatic and chromatic parameters of Sauvignon Blanc wines. It can be concluded that only treatments with 5% sulfurous acid resulted in complete alcoholic fermentation, i.e., dry wines and lowest absorbance in wines. Addition of reducing agents containing ascorbic acid and tannins increased total phenols, antioxidant activity and color intensity in wines. Significant differences in the aromatic profile of wines were noticed due to the difference in the total and individual aromatic compound concentrations. Based on the achieved results, a combination of potassium metabisulfite, ascorbic acid, gallotannins and ellagitannins positively influenced the concentrations and OAVs of some individual and total aldehydes, esters and terpenes with medial concentration of total SO_2_ in wine.

## Figures and Tables

**Table 1:** Physicochemical properties, total phenols and antioxidant capacity of Sauvignon Blanc wines.

Parameter	A	B	C	D	E	F
Alcohol (%, *v*/*v*)	13. 7± 0.0	13.6 ± 0.0	13.6 ± 0.0	13.5 ± 0.0	13.7 ± 0.0	13.6 ± 0.0
Residual sugars (g/L)	2.8 ± 0.1 ^e^	2.7 ± 0.1 ^e^	5.8 ± 0.1 ^b^	6.2 ± 0.1 ^a^	5.3 ± 0.1 ^d^	5.6 ± 0.1 ^c^
Sugar-free extract (g/L)	18.0 ± 0.1 ^d^	18.9 ± 0.1 ^a^	18.4 ± 0.1 ^b^	17.7 ± 0.1 ^f^	17.9 ± 0.1 ^e^	18.3 ± 0.1 ^c^
Total acidity * (g/L)	5.8 ± 0.1 ^e^	6.6 ± 0.1 ^a^	6.0 ± 0.1 ^d^	5.9 ± 0.0 ^e^	6.2 ± 0.1 ^c^	6.4 ± 0.0 ^b^
Volatile acidity ** (g/L)	0.60 ± 0.0 ^c^	0.54 ± 0.0 ^d^	0.65 ± 0.0 ^b^	0.70 ± 0.0 ^a^	0.65 ± 0.0 ^b^	0.59 ± 0.0 ^c^
pH	3.3 ± 0.0	3.3 ± 0.0	3.3 ± 0.0	3.3 ± 0.0	3.3 ± 0.0	3.3 ± 0.0
SO2 bound (mg/L)	37.0 ± 0.5 ^f^	40.0 ± 0.5 ^e^	59.0 ± 0.5 ^a^	55.0 ± 0.5 ^c^	57.0 ± 0.5 ^b^	45.0 ± 0.5 ^d^
SO2 total (mg/L)	37.0 ± 0.05 ^f^	40.0 ± 0.0 ^e^	59.0 ± 0.5 ^a^	55.0 ± 0.5 ^c^	57.0 ± 0.5 ^b^	45.0 ± 0.5 ^d^
Ash (g/L)	1.71 ± 0.0 ^d^	1.74 ± 0.0 ^b^	1.73 ± 0.0 ^c^	1.72 ± 0.0 ^c^	1.72 ± 0.0 ^c^	1.78 ± 0.0 ^a^
Total phenols (mg/L GAE)	241.69 ± 0.0 ^f^	250.75 ± 0.0 ^d^	275.63 ± 0.0 ^b^	267.8 ± 0.0 ^c^	278.02 ± 0.1 ^a^	247.77 ± 0.0 ^e^
ABTS (µM/L TE)	1.26 ± 0.0 ^d^	1.34 ± 0.0 ^c,d^	1.44 ± 0.0 ^a,b^	1.42 ± 0.0 ^a,b,c^	1.50 ± 0.0 ^a^	1.37 ± 0.0 ^b,c^

* tartaric acid and ** acetic acid equivalents. ABTS = antioxidant capacity (μmol of Trolox equivalents/L) by 2,20-azinobis-(3-ethylbenzothiazoline-6-sulfonic acid) radical cation (ABTS^+^). Concentrations expressed as mean ± standard deviation (n = 3). Means with different superscript letters in the same row differ significantly (*p* ≤ 0.05).

**Table 2:** Concentration of volatile compounds in Sauvignon Blanc wines (mg/L).

Compounds	Q_Ion_	R_t_	A			B			C			D			E			F		
**Aldehydes**																				
Hexanal	44	21.93	460.00	±	0.71 ^b^	466.86	±	0.49 ^a^	333.28	±	1.22 ^f^	381.17	±	1.12 ^e^	427.69	±	0.37 ^c^	397.71	±	0.79 ^d^
2-Pentanal	55	24.13	12.50	±	0.26 ^e^	16.52	±	0.02 ^b^	15.47	±	0.27 ^c^	25.06	±	0.15 ^a^	14.43	±	0.01 ^d^	14.00	±	0.11 ^d^
2-Octenal (g/L)	41	44.71	1.15	±	0.00 ^e^	1.18	±	0.01 ^e^	2.72	±	0.00 ^b^	2.83	±	0.00 ^a^	1.79	±	0.00 ^d^	1.94	±	0.00 ^c^
Decanal	43	48.97	0.88	±	0.01 ^f^	2.77	±	0.08 ^d^	1.96	±	0.03 ^e^	5.47	±	0.06 ^a^	3.44	±	0.02 ^c^	4.93	±	0.03 ^b^
Benzaldehyde	106	51.01	17.60	±	0.01 ^b^	21.04	±	0.12 ^a^	13.24	±	0.07 ^e^	17.06	±	0.15 ^c^	11.89	±	0.10 ^f^	14.06	±	0.23 ^d^
**Esters**																				
Isobutyl acetate	43	16.83	54.17	±	0.02 ^d^	44.41	±	0.06 ^e^	80.56	±	0.16 ^b^	83.06	±	0.12 ^a^	57.69	±	0.38 ^c^	57.34	±	0.23 ^c^
Ethyl butanoate	71	18.03	281.50	±	0.57 ^c^	236.08	±	1.29 ^d^	340.64	±	4.94 ^b^	355.40	±	13.30 ^a^	283.54	±	0.70 ^c^	284.57	±	0.78 ^c^
Ethyl-2-methylbutanoate	57	19.10	18.41	±	0.03 ^e^	21.82	±	0.05 ^b^	17.63	±	0.01 ^f^	19.06	±	0.01 ^d^	22.09	±	0.04 ^a^	19.36	±	0.06 ^c^
Ethyl-3-methylbutanoate	88	20.11	26.56	±	0.01 ^b^	28.37	±	0.05 ^a^	22.77	±	0.01 ^d^	24.94	±	0.13 ^c^	29.14	±	0.22 ^a^	25.34	±	0.68 ^b,c^
Isoamyl acetate (g/L)	43	23.51	1.69	±	0.00 ^d^	1.06	±	0.00 ^e^	2.23	±	0.00 ^b^	2.28	±	0.01 ^a^	1.70	±	0.00 ^d^	1.78	±	0.75 ^c^
Ethyl hexanoate	88	31.12	316.08	±	0.67 ^e^	219.50	±	0.69 ^f^	545.54	±	0.86 ^b^	563.51	±	2.32 ^a^	357.84	±	2.16 ^d^	385.87	±	0.41 ^c^
Ethyl lactate	45	38.67	18.75	±	0.06 ^b^	19.76	±	0.14 ^a^	13.95	±	0.07 ^e^	13.45	±	0.04 ^f^	15.95	±	0.09 ^d^	18.40	±	0.05 ^c^
Ethyl-2-hydroxy-3-methylbutanoate	73	44.52	417.86	±	1.00 ^e^	293.81	±	0.65 ^f^	672.94	±	2.22 ^b^	697.27	±	5.46 ^a^	440.19	±	0.74 ^d^	476.98	±	1.46 ^c^
Ethyl octanoate	88	44.69	585.83	±	0.42 ^e^	409.07	±	0.29 ^f^	943.26	±	1.91 ^b^	979.04	±	10.95 ^a^	620.44	±	1.70 ^d^	671.91	±	2.21 ^c^
Isobutyl lactate	45	53.70	87.93	±	0.16 ^b^	114.87	±	0.41 ^a^	72.30	±	0.11 ^c^	71.72	±	0.16 ^c^	68.57	±	0.01 ^d^	57.84	±	0.64 ^e^
Isoamyl lactate	45	53.70	31.66	±	0.03 ^b^	42.64	±	0.04 ^a^	31.50	±	0.07 ^b^	27.28	±	0.02 ^c^	23.08	±	0.06 ^d^	22.35	±	0.04 ^e^
Ethyl furoate	95	56.88	15.88	±	0.01 ^e^	10.89	±	0.03 ^f^	25.54	±	0.01 ^b^	28.11	±	0.37 ^a^	18.16	±	0.03 ^d^	19.31	±	0.01 ^c^
Ethyl decanoate	88	57.31	526.86	±	0.01 ^e^	365.89	±	0.04 ^f^	880.98	±	2.46 ^b^	935.10	±	0.78 ^a^	591.66	±	1.42 ^d^	659.37	±	1.50 ^c^
Diethyl succinate (g/L)	101	59.67	1.15	±	0.04 ^b^	1.30	±	0.02 ^a^	0.88	±	0.00 ^d^	0.99	±	0.02 ^c^	1.12	±	0.01 ^b^	1.23	±	0.70 ^a^
Ethylmethyl succinate	101	61.00	38.31	±	0.01 ^e^	26.85	±	0.04 ^f^	61.78	±	0.02 ^b^	65.76	±	0.02 ^a^	42.41	±	0.01 ^d^	47.34	±	0.08 ^c^
Diethyl glutarate	143	65.93	0.60	±	0.01 ^d^	1.11	±	0.01 ^a^	0.44	±	0.01 ^e^	0.66	±	0.01 ^c^	0.98	±	0.01 ^b^	0.70	±	0.01 ^c^
Methyl-3-hydroxyoctanoate	103	77.10	0.85	±	0.04 ^b^	2.60	±	0.11 ^a^	0.32	±	0.01 ^d^	0.89	±	0.02 ^b^	0.67	±	0.02 ^c^	0.99	±	0.01 ^b^
Diethyl malonate	117	79.86	0.36	±	0.03 ^c^	0.73	±	0.03 ^a^	0.47	±	0.02 ^b^	0.31	±	0.01 ^c^	0.35	±	0.02 ^c^	0.32	±	0.02 ^c^
Ethyl hexadecanoate	88	88.41	8.29	±	0.19 ^d,e^	7.73	±	0.09 ^e^	16.30	±	0.07 ^b^	21.40	±	0.60 ^a^	9.12	±	0.23 ^d^	12.18	±	0.21 ^c^
Ethyl hydrogensuccinate (g/L)	101	96.60	2.26	±	0.00 ^b,c^	2.60	±	0.00 ^a^	1.90	±	0.00 ^e^	2.30	±	0.00 ^b^	2.21	±	0.02 ^cd^	2.18	±	0.02 ^d^
Ethyl linoleate	67	101.16	2.17	±	0.02 ^b^	3.81	±	0.06 ^a^	1.79	±	0.04 ^c^	1.54	±	0.07 ^d^	1.27	±	0.01 ^e^	1.24	±	0.01 ^e^
**Alcohols**																				
1-Butanol	56	24.66	28.21	±	0.04 ^e^	28.94	±	0.10 ^e^	88.67	±	0.49 ^b^	97.05	±	0.04 ^a^	74.54	±	0.75 ^d^	78.43	±	0.15 ^c^
2-Methyl-1-butanol (g/L)	55	28.96	31.40	±	0.06 ^a^	32.18	±	0.02 ^a^	26.45	±	0.06 ^a^	30.92	±	0.05 ^a^	31.01	±	0.08 ^a^	30.49	±	0.01 ^a^
Isoamyl alcohol (g/L)	41	29.03	33.53	±	0.03 ^f^	60.75	±	0.03 ^a^	49.10	±	0.07 ^e^	58.55	±	0.06 ^f^	59.25	±	0.03 ^b^	57.01	±	0.10 ^d^
1-Pentanol	42	32.20	2.07	±	0.01 ^a^	1.59	±	0.02 ^b^	0.77	±	0.06 ^d^	1.07	±	0.03 ^c^	0.12	±	0.02 ^e^	0.24	±	0.01 ^e^
4-Methyl-1-pentanol	56	36.70	101.77	±	0.08 ^b^	79.66	±	0.48 ^e^	86.77	±	0.08 ^d^	101.36	±	0.25 ^b^	117.60	±	0.02 ^a^	97.76	±	0.29 ^c^
2-Heptanol	45	36.90	1.09	±	0.01 ^b^	0.15	±	0.01 ^e^	1.69	±	0.02 ^a^	0.17	±	0.01 ^e^	0.59	±	0.02 ^c^	0.26	±	0.01 ^d^
2-Pentene-1-ol	57	37.10	58.20	±	0.01 ^d^	44.48	±	0.06 ^f^	54.27	±	0.39 ^e^	64.24	±	0.03 ^b^	69.45	±	0.64 ^a^	59.94	±	0.05 ^c^
3-Methyl-1-pentanol	56	37.56	227.87	±	0.16 ^d^	180.03	±	0.30 ^f^	218.65	±	0.62 ^e^	252.42	±	0.37 ^b^	277.34	±	0.70 ^a^	240.88	±	0.25 ^c^
1-Hexanol (g/L)	56	39.88	1.05	±	0.01 ^b^	1.12	±	0.01 ^a^	0.68	±	0.00 ^f^	0.77	±	0.01 ^e^	0.83	±	0.00 ^d^	0.91	±	0.00 ^c^
3-Hexene-1-ol, trans	41	39.88	32.60	±	0.14 ^d^	31.50	±	0.11 ^e^	56.16	±	0.04 ^b^	59.18	±	0.21 ^a^	52.15	±	0.29 ^c^	31.04	±	0.39 ^e^
3-Hexene-1-ol, cis	67	41.76	16.38	±	0.04 ^e^	12.39	±	0.12 ^f^	26.63	±	0.28 ^b^	28.17	±	0.21 ^a^	19.77	±	0.07 ^d^	21.10	±	0.06 ^c^
Cyclohexanol	57	41.86	1.50	±	0.11 ^a^	1.29	±	0.01 ^b^	0.92	±	0.02 ^c^	0.80	±	0.02 ^c,d^	1.17	±	0.01 ^b^	0.73	±	0.03 ^d^
2-Hexene-1-ol, trans	57	42.72	9.21	±	0.25 ^a^	8.06	±	0.03 ^b^	4.81	±	0.05 ^d^	5.46	±	0.15 ^d^	6.24	±	0.37 ^c^	7.55	±	0.11 ^b^
1-Octen-3-ol	57	45.53	39.36	±	0.52 ^b^	44.70	±	0.97 ^a^	25.01	±	1.03 ^e^	30.37	±	0.13 ^d^	35.43	±	0.65 ^c^	33.77	±	0.66 ^c^
1-Heptanol	70	45.93	1.13	±	0.01 ^d^	0.82	±	0.04 ^e^	2.49	±	0.05 ^a^	2.59	±	0.06 ^a^	1.60	±	0.01 ^b^	1.39	±	0.04 ^c^
2-Ethyl-1-hexanol	57	48.07	0.81	±	0.02 ^a^	0.51	±	0.03 ^c^	0.64	±	0.04 ^b^	0.84	±	0.03 ^a^	0.66	±	0.01 ^b^	0.39	±	0.03 ^d^
2,6-Dimethyl-4-heptanol	69	51.86	5.27	±	0.04 ^a,b^	3.86	±	0.11 ^e^	4.42	±	0.03 ^d^	4.89	±	0.01 ^c^	5.03	±	0.05 ^b,c^	5.29	±	0.03 ^a^
1-Octanol	56	52.32	5.43	±	0.01 ^c^	5.14	±	0.01 ^d^	5.59	±	0.04 ^b^	5.76	±	0.04 ^b^	5.63	±	0.05 ^b^	6.04	±	0.04 ^a^
2,3-Butanediol	45	53.52	11.09	±	0.02 ^c^	5.58	±	0.01 ^e^	7.38	±	0.05 ^d^	5.56	±	0.01 ^e^	11.86	±	0.08 ^b^	15.91	±	0.13 ^a^
2-Octen-1-ol	57	56.04	0.11	±	0.01 ^c^	0.23	±	0.02 ^b^	0.24	±	0.00 ^b^	0.25	±	0.01 ^b^	0.10	±	0.01 ^c^	0.34	±	0.01 ^a^
1-Decanol	55	64.27	4.44	±	0.01 ^b^	5.07	±	0.02 ^a^	3.76	±	0.04 ^e^	4.27	±	0.02 ^c^	4.31	±	0.02 ^c^	4.06	±	0.01 ^d^
Phenylethyl Alcohol (g/L)	91	72.78	32.93	±	0.44 ^b,c^	33.42	±	0.10 ^a^	29.82	±	0.02 ^d^	33.39	±	0.07 ^ab^	33.99	±	0.08 ^a^	31.97	±	0.11 ^c^
2-Pentadecanol	45	74.97	3.20	±	0.01 ^b^	2.80	±	0.13 ^c^	2.82	±	0.01 ^c^	2.87	±	0.01 ^c^	2.94	±	0.03 ^c^	4.21	±	0.02 ^a^
1-Octadecanol	83	104.43	3.55	±	0.10 ^a^	2.60	±	0.07 ^b^	2.73	±	0.01 ^b^	2.37	±	0.13 ^b^	2.42	±	0.04 ^b^	2.40	±	0.06 ^b^
**Terpenes**																				
γ-Terpinene	93	32.03	25.39	±	0.06 ^b^	28.55	±	0.35 ^a^	20.10	±	0.16 ^d^	22.19	±	0.08 ^c^	25.24	±	0.38 ^b^	28.16	±	0.02 ^a^
Tetrahydrolinalool	73	44.32	53.27	±	0.09 ^b^	59.79	±	0.24 ^a^	24.07	±	0.23 ^f^	29.26	±	0.09 ^e^	39.94	±	0.12 ^c^	34.15	±	0.23 ^d^
Linalyl formate	69	46.98	15.02	±	0.09 ^b^	15.70	±	0.09 ^a^	12.21	±	0.14 ^c^	14.95	±	0.28 ^b^	14.70	±	0.33 ^b^	14.99	±	0.11 ^b^
Linalool	71	51.72	46.63	±	0.09 ^b^	52.94	±	0.76 ^a^	35.01	±	0.40 ^e^	39.40	±	0.07 ^d^	42.84	±	0.03 ^c^	40.47	±	0.12 ^d^
Terpinene-4-ol	71	55.47	37.72	±	0.35 ^b^	40.44	±	0.40 ^a^	36.00	±	0.86 ^b,c^	42.16	±	0.42 ^a^	37.18	±	0.28 ^b^	35.04	±	0.04 ^c^
Hotrienol	71	55.74	51.16	±	0.72 ^c^	55.72	±	0.21 ^b^	51.14	±	0.02 ^c^	58.02	±	0.16 ^a^	51.36	±	0.24 ^c^	48.23	±	**0.30 ^d^**
α-Terpineol	59	60.92	4.93	±	0.01 ^a^	4.95	±	0.03 ^a^	4.02	±	0.06 ^c^	4.58	±	0.06 ^b^	4.99	±	0.05 ^a^	4.53	±	**0.05 ^a^**
Linalool oxide pyran	68	63.38	5.52	±	0.01 ^b^	4.38	±	0.06 ^c^	5.56	±	0.04 ^a,b^	5.69	±	0.01 ^a^	4.39	±	0.01 ^c^	5.59	±	**0.02 ^a,b^**
Citronellol	69	64.53	10.98	±	0.06 ^b^	19.10	±	0.11 ^a^	8.10	±	0.04 ^d^	11.34	±	0.08 ^b^	10.21	±	0.13 ^c^	10.04	±	**0.11 ^c^**
Nerol	69	66.54	159.66	±	0.74 ^c^	171.77	±	0.69 ^a^	150.52	±	0.23 ^d^	174.11	±	0.78 ^a^	164.61	±	1.48 ^b^	157.98	±	**0.03 ^c^**
Geraniol	69	68.99	49.29	±	0.52 ^e^	40.18	±	0.01 ^f^	81.83	±	0.02 ^b^	83.83	±	0.30 ^a^	55.35	±	0.54 ^d^	57.61	±	**0.28 ^c^**
Terpendiol II	67	74.09	39.80	±	0.21 ^b^	22.34	±	0.22 ^d^	41.87	±	0.18 ^a^	41.05	±	0.36 ^a^	34.72	±	0.14 ^c^	41.99	±	**0.09 ^a^**
6,7-Dihydro-7-hydroxylinalool	71	75.47	3.39	±	0.18 ^b^	2.68	±	0.04 ^c^	4.24	±	0.20 ^a^	3.99	±	0.16 ^a^	2.31	±	0.10 ^c^	3.24	±	**0.10 ^b^**
Neralidol	69	78.55	18.49	±	0.06 ^a^	12.01	±	0.08 ^d^	17.22	±	0.14 ^c^	17.94	±	0.04 ^b^	12.28	±	0.11 ^d^	18.68	±	**0.01 ^a^**
Geranyl acetate	69	91.21	7.57	±	0.02 ^c^	7.03	±	0.06 ^c^	4.54	±	0.18 ^d^	9.24	±	0.08 ^b^	9.05	±	0.13 ^b^	11.19	±	**0.26 ^a^**
8-Hidroxylinalool	43	91.41	4.51	±	0.01 ^d^	1.81	±	0.01 ^e^	7.50	±	0.21 ^c^	13.22	±	0.47 ^a^	4.62	±	0.11 ^d^	12.02	±	**0.02 ^b^**
**Volatile phenols**																				
Benzaacetaldehyde	91	58.28	0.00	±	0.00	0.00	±	0.00	3.41	±	0.01 ^a^	2.78	±	0.02 ^c^	2.82	±	0.01 ^b^	1.08	±	**0.01 ^d^**
Benzylalcohol	78	70.92	4.52	±	0.02 ^a^	4.79	±	0.02 ^a^	3.57	±	0.04 ^c^	3.99	±	0.18 ^b^	3.38	±	0.06 ^c^	3.74	±	**0.06 ^c^**
4-Ethylguaiacol	85	78.84	3.95	±	0.04 ^a^	0.00	±	0.00	3.16	±	0.01 ^b^	0.00	±	0.00	4.01	±	0.04 ^a^	0.00	±	**0.00**
Tyrosol	107	97.21	23.88	±	0.08 ^a^	6.32	±	0.01 ^e^	18.15	±	0.01 ^b^	15.81	±	0.02 ^f^	16.82	±	0.40 ^c^	11.12	±	**0.31 ^d^**
Vanilin	151	102.78	4.71	±	0.01 ^a^	4.50	±	0.01 ^b^	4.59	±	0.02 ^b^	5.14	±	0.06 ^b^	4.43	±	0.12 ^b^	4.52	±	**0.04 ^b^**
Methyl vanillate	151	104.17	2.75	±	0.01 ^c^	3.21	±	0.03 ^a^	2.61	±	0.06 ^c^	2.94	±	0.03 ^b^	2.47	±	0.01 ^d^	2.28	±	**0.01 ^e^**
Ethyl vanillate	151	106.62	3.06	±	0.03 ^b^	5.89	±	0.06 ^a^	0.45	±	0.02 ^c^	0.00	±	0.00	0.48	±	0.02 ^c^	0.00	±	**0.00**
Homovanillyl alcohol	137	115.17	1.40	±	0.01 ^b^	0.37	±	0.01 ^e^	1.55	±	0.04 ^a^	1.27	±	0.01 ^c^	0.38	±	0.01 ^e^	0.77	±	**0.04 ^d^**
**Fatty acids**																				
Propanoic acid	74	51.97	29.95	±	0.04 ^e^	20.47	±	0.02 ^f^	33.91	±	0.10 ^c^	32.20	±	0.07 ^d^	38.42	±	0.01 ^a^	36.09	±	**0.04 ^b^**
2-Methylpropionic acid	43	54.57	7.77	±	0.01 ^b^	6.30	±	0.02 ^c^	5.09	±	0.02 ^e^	10.96	±	0.13 ^a^	6.23	±	0.03 ^c^	5.58	±	**0.03 ^d^**
Isovaleric acid	60	60.48	4.41	±	0.01 ^d^	5.30	±	0.07 ^c^	5.53	±	0.05 ^c^	8.24	±	0.08 ^b^	11.02	±	0.09 ^a^	5.46	±	**0.08 ^c^**
Heptanoic acid	60	75.04	8.90	±	0.08 ^d^	8.23	±	0.06 ^e^	8.48	±	0.02 ^e^	10.63	±	0.05 ^b^	9.69	±	0.06 ^c^	11.22	±	**0.16 ^a^**
Octanoic acid	60	80.35	8.50	±	0.01 ^a^	6.57	±	0.02 ^c^	2.38	±	0.05 ^e^	7.33	±	0.04 ^b^	4.26	±	0.17 ^d^	4.40	±	**0.01 ^d^**
**Miscellaneous**																				
4-Methyl--2-penten-2-one	55	24.45	21.86	±	0.02 ^e^	22.04	±	0.04 ^e^	41.22	±	0.24 ^b^	47.77	±	0.10 ^a^	31.77	±	0.62 ^d^	34.05	±	**0.11 ^c^**
Acetoin	45	34.89	4.97	±	0.08 ^b^	5.61	±	0.02 ^a^	4.95	±	0.04 ^b^	4.53	±	0.03 ^c^	4.21	±	0.03 ^d^	4.88	±	**0.01 ^b^**
6-Methyl-5-hepten-2-on	43	38.52	150.75	±	0.13 ^e^	164.77	±	0.71 ^d^	149.79	±	0.05 ^e^	181.68	±	0.64 ^c^	187.90	±	0.11 ^b^	212.40	±	**0.44 ^a^**
N-Ethylacetamide	43	57.77	3.87	±	0.21 ^d^	5.05	±	0.02 ^c^	2.51	±	0.01 ^e^	7.38	±	0.04 ^a^	5.77	±	0.01 ^b^	2.48	±	**0.03 ^e^**
Dihydro-2-methyl-3(2H)furanone	43	62.67	2.76	±	0.00 ^a^	2.25	±	0.02 ^b^	2.70	±	0.01 ^a^	1.64	±	0.03 ^c^	0.13	±	0.01 ^e^	0.94	±	**0.05 ^d^**
ß-Damascenone	69	68.11	6.64	±	0.04 ^b^	6.80	±	0.15 ^b^	5.70	±	0.01 ^d^	5.77	±	0.01 ^d^	6.10	±	0.07 ^c^	7.44	±	**0.04 ^a^**
4-(Methylthio)-1-butanol	61	69.56	289.97	±	0.33 ^e^	210.35	±	1.75 ^f^	490.34	±	6.85 ^b^	507.99	±	0.32 ^a^	321.50	±	0.74 ^d^	340.92	±	**1.08 ^c^**
γ-Carboethoxy-γ-butyrolactone	85	85.57	109.90	±	0.11 ^c^	15.04	±	0.03 ^e^	29.13	±	0.78 ^e^	66.23	±	0.81 ^d^	390.76	±	10.70 ^a^	169.20	±	**6.33 ^b^**
N-(2-phenylethyl) acetamide	104	106.27	2.82	±	0.01 ^b^	2.79	±	0.04 ^bc^	3.08	±	0.01 ^a^	2.84	±	0.04 ^b^	2.59	±	0.04 ^cd^	2.50	±	**0.06 ^d^**

Concentrations expressed as mean ± standard deviation (n = 3). Means with different superscript letters in the same row differ significantly (*p* ≤ 0.05). Legend: Q_Ion_ = Ion quantifier; R_t_ = retention time.

**Table 3:** Odor activity values (OAV), relative odor contribution (ROC) and odor thresholds (OTH) of aromatic compounds with OAV > 1 in Sauvignon Blanc wines.

Compounds	OTH (mg/L)	Odor Descriptor	OAV					ROC (%)						
			A	B	C	D	E	F	A	B	C	D	E	F
2-Octenal	0.003 ^1^	Green, nut, fat	384.00	394.64	905.60	942.40	597.70	647.29	46.82	49.72	69.61	69.13	60.37	60.87
Hexanal	0.045 ^1^	Green, grass	102.22	103.75	74.06	84.70	95.04	88.38	12.46	13.07	5.69	6.21	9.60	8.31
Isoamyl acetate	0.030 ^2^	Banana	56.42	35.24	74.45	76.01	56.76	59.47	6.88	4.40	5.72	5.57	5.73	5.59
Ethyl octanoate	0.580 ^3^	Sweet, floral, fruity, pear	1.01	<1	1.62	1.69	1.07	1.16	0.12	-	0.12	0.12	0.11	0.11
Ethyl decanoate	0.200 ^3^	Floral	2.63	1.83	4.40	4.67	2.96	3.30	0.32	0.23	0.33	0.34	0.30	0.31
Ethyl hexanoate	0.014 ^2^	Fruity, green apple, banana	22.56	15.68	38.97	40.25	25.56	27.56	2.75	1.98	2.99	2.95	2.58	2.59
Ethyl butanoate	0.020 ^4^	Pineapple, apple	14.07	11.80	17.03	17.77	14.18	14.23	1.71	1.49	1.30	1.30	1.43	1.34
Isoamyl lactate	0.0016 ^5^	Fruit, apple, banana	19.79	26.65	19.69	17.05	14.42	13.97	2.41	3.36	1.51	1.25	1.45	1.31
Ethyl-3-methylbutanoate	0.003 ^4^	Fruity, pineapple	8.85	9.46	7.59	8.31	9.71	8.45	1.07	1.19	0.58	0.61	0.98	0.79
Ethyl-2-methylbutanoate	0.018 ^4^	Apple	1.02	1.21	<1	1.06	1.23	1.08	0.12	0.15	-	0.07	0.12	0.10
Ethyl hexadecanoate	0.0015 ^6^	Fruity, wax	5.53	5.15	10.87	14.27	6.08	8.12	0.67	0.65	0.83	1.04	0.61	0.76
Phenylethyl alcohol	14 ^2^	Floral, rose, honey	2.35	2.39	2.13	2.38	2.42	2.28	0.28	0.30	0.16	0.17	0.24	0.21
2-Methyl-1-butanol	30 ^7^	Whiskey, burnt, nail polish	1.05	1.07	<1	1.03	1.03	1.02	0.13	0.13	-	0.07	0.10	0.09
1-Octen-3-ol	0.001 ^8^	Mushroom	39.36	44.70	25.01	30.37	35.43	33.77	4.80	5.63	1.92	2.22	3.58	3.17
Geraniol	0.020 ^2^	Citrus, citric fruit	24.64	2.01	4.09	4.19	2.77	2.88	3.00	0.25	0.31	0.31	0.28	0.27
Linalool	0.025 ^2^	Citrus, floral, sweet	1.86	2.12	1.40	1.58	1.71	1.62	0.23	0.27	0.11	0.11	0.17	0.15
ß-Damascenone	0.00005 ^9^	Sweet, fruity, floral, honey	132.80	136.00	114.00	115.40	122.00	148.80	16.19	17.13	8.76	8.46	12.32	13.99

Odor threshold values (OTH) from the literature: ^1^ [47], ^2^ [48], ^3^ [49], ^4^ [50], ^5^ [51], ^6^ [52], _7_ [53], ^8^ [54], ^9^ [55].
